# Potential of Chlorogenic Acid in the Management of Metabolic Dysfunction-Associated Steatotic Liver Disease (MASLD): Animal Studies and Clinical Trials—A Narrative Review

**DOI:** 10.3390/metabo14060346

**Published:** 2024-06-20

**Authors:** Agnieszka Ziółkiewicz, Przemysław Niziński, Jakub Soja, Tomasz Oniszczuk, Maciej Combrzyński, Adrianna Kondracka, Anna Oniszczuk

**Affiliations:** 1 Department of Inorganic Chemistry, Medical University of Lublin, Dr Witolda Chodźki 4a, 20-093 Lublin, Poland; ziolp@o2.pl (A.Z.); anna.oniszczuk@umlub.pl (A.O.); 2 Department of Pharmacology, Medical University of Lublin, Radziwiłłowska 11, 20-080 Lublin, Poland; 3 Department of Thermal Technology and Food Process Engineering, University of Life Sciences in Lublin, Głęboka 31, 20-612 Lublin, Poland; jakub.soja@up.lublin.pl (J.S.); tomasz.oniszczuk@up.lublin.pl (T.O.); maciej.combrzynski@up.lublin.pl (M.C.); 4 Department of Obstetrics and Pathology of Pregnancy, Medical University of Lublin, 20-081 Lublin, Poland; adriannnakondracka@umlub.pl

**Keywords:** chlorogenic acid, metabolic dysfunction-associated steatotic liver disease (MASLD), hepatic steatosis, animal studies, clinical trials

## Abstract

Chlorogenic acid (CGA) is a natural polyphenol found in coffee, tea, vegetables, and fruits. It exhibits strong antioxidant activity and possesses several other biological properties, including anti-inflammatory effects, antimicrobial activity, and insulin-sensitizing properties. Moreover, it may improve lipid and glucose metabolism. This review summarizes the available information on the therapeutic effect of CGA in metabolic dysfunction-associated steatotic liver disease (MASLD). As the literature search engine, the browsers in the PubMed, Scopus, Web of Science databases, and ClinicalTrials.gov register were used. Animal trials and clinical studies suggest that CGA has promising therapeutic potential in treating MASLD and hepatic steatosis. Its mechanisms of action include antioxidant, anti-inflammatory, and anti-apoptotic effects via the activation of the Nrf2 signaling pathway and the inhibition of the TLR4/NF-κB signaling cascade. Furthermore, the alleviation of liver disease by CGA also involves other important molecules such as AMPK and important physiological processes such as the intestinal barrier and gut microbiota. Nevertheless, the specific target cell and key molecule to which CGA is directed remain unidentified and require further study.

## 1. Introduction

The liver performs various functions, such as lipid and glucose metabolism. Glycogenolysis, glycogen synthesis, and gluconeogenesis are among its roles in carbohydrate metabolism. The organ also contributes to lipid and lipoprotein metabolism by synthesizing apolipoproteins, triglycerides, and cholesterol, as well as folding lipoproteins and eliminating cholesterol. Bile acids (BAs) represent a significant component of bile, a digestive fluid produced by the liver. BAs are synthesized from cholesterol in the liver via two principal pathways. They are stored in the gallbladder and released into the duodenum in response to the ingestion of food. BAs play a pivotal role in metabolic homeostasis [1]. The primary function of bile acids is to emulsify, thereby facilitating the absorption of lipids and water-insoluble vitamins [2]. It has been demonstrated that BAs also function as signaling molecules, activating the farnesoid X receptor (FXR) and membrane Takeda G protein-coupled receptor 5 (TGR5) [3].

Nonalcoholic fatty liver disease (NAFLD) is a prevalent chronic liver disease worldwide. It is the result of the excessive accumulation of large fat droplets in hepatocytes (hepatic steatosis) [4]. The term ‘nonalcoholic fatty liver disease’ was first used by Schaner and Thaler in 1986, but the word ‘nonalcoholic’ has several drawbacks and can be considered stigmatizing [5]. Furthermore, the recommendation of complete abstinence from alcohol may leave a grey area for patients who consume alcohol in moderation and may suffer from both alcoholic and metabolic diseases. However, some individuals perceive the term ‘steatosis’ to have a negative connotation [5,6], so the three large associations dealing with liver diseases led to a Delphi process to create a new nomenclature. The chosen name to replace NAFLD is MASLD—metabolic dysfunction-associated steatotic liver disease [7,8,9].

MASLD is frequently associated with other disorders such as cardiovascular and metabolic diseases, diabetes, and obesity [10]. It is estimated that in the next two decades, metabolic dysfunction-associated steatohepatitis (MASH) will become a major cause of hepatocellular carcinoma and end-stage liver failure [4]. The increasing incidence of type 2 diabetes, cardiovascular disease, obesity, and MASLD is largely attributed to a sedentary lifestyle and the excessive consumption of highly processed foods [9]. There is growing evidence that various dietary components may contribute to the development of MASLD or, conversely, help prevent the disease [11]. For many individuals, coffee is a significant component of their daily dietary intake. It is one of the most commonly consumed beverages globally and contains numerous biologically active compounds. There is ample evidence to suggest that consuming 1–2 cups of coffee per day can reduce the risk of diseases such as type 2 diabetes and Parkinson’s disease [12]. Additionally, experimental studies have indicated a preventive and inhibitory effect of coffee on MASLD [13]. Chlorogenic acid (CGA) is one of the primary active compounds found in coffee along with caffeine. Its content in a standard 200–240 mL cup of coffee is estimated to range from 70 to 350 mg [13].

Chlorogenic acid is an ester of caffeic acid and quinic acid. It exhibits strong antioxidant activity and possesses several other biological properties, including anti-inflammatory effects, antimicrobial activity, and insulin-sensitizing properties [14]. Moreover, it may improve lipid and glucose metabolism by facilitating the removal of triglycerides and free fatty acids, as well as enhancing insulin sensitivity [14]. CGA has been shown to have cholesterol-lowering effects and can alleviate oxidative stress induced by a high-fat diet. A recent study also demonstrated that CGA can alleviate hepatic steatosis and inhibit fatty acid synthesis [15].

This review summarizes the available information on the therapeutic effect of chlorogenic acid in the management of metabolic dysfunction-associated steatotic liver disease. As the literature search engine, the browsers in the PubMed, Scopus, Web of Science databases, and ClinicalTrials.gov register were used. The following inquiries were used: ‘NAFLD and polyphenols’, ‘NAFLD and chlorogenic acid’, ‘NAFLD and clinical trials’, ‘NAFLD and animal models’, ‘chlorogenic acid and hepatic steatosis’, ‘chlorogenic acid and nonalcoholic fatty liver disease’, ‘MASLD and polyphenols’, ‘MASLD and chloro-genic acid’, ‘MASLD and clinical trials’, ‘MASLD and animal models’, ‘chlorogenic acid and hepatic steatosis’, and ‘chlorogenic acid and metabolic dysfunction-associated steatotic liver disease’. The documents published from 1990 to April 2024 were included.

## 2. General Overview of MASLD and Connection with MetS

**Figure 1:**
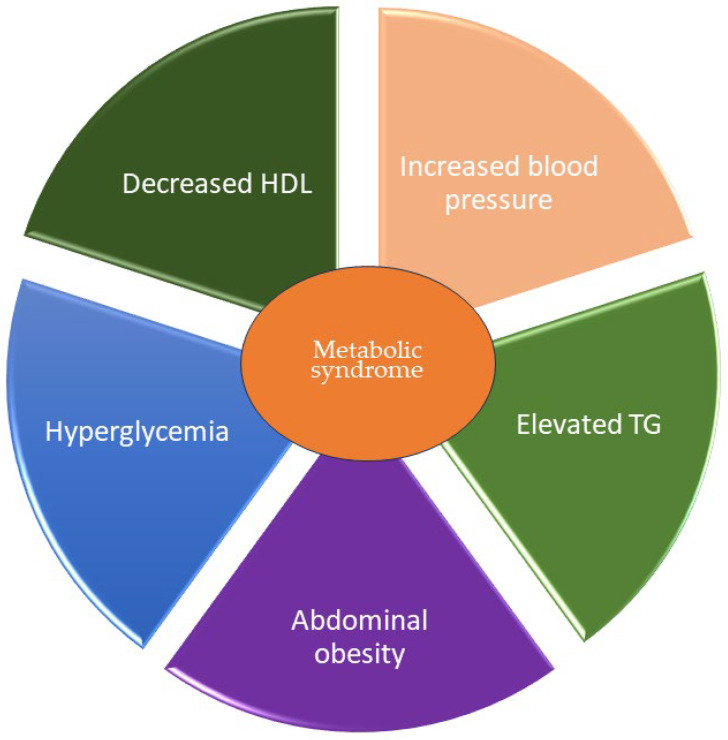
Schematic representation of main symptoms of metabolic syndrome. Abbreviations: HDL—high-density lipoproteins, TG—triglycerides.

Metabolic dysfunction-associated steatotic liver disease, formerly known as nonalcoholic fatty liver disease, is a common reason of chronic liver disorder worldwide [7]. A meta-analysis by Riazi et al. 2022 reports that the prevalence of MASLD has notably risen from about 25% before 2005 to nearly 38% in 2016 and later. The incidence of MASLD was found to be significantly higher in men than in women (about 71 and 30 cases per 1000 person-years, respectively) [16]. MASLD is deemed as a hepatic manifestation (or rather, a wide spectrum of symptoms) of metabolic syndrome (MetS) [17]. The term ‘metabolic syndrome’ comprises a number of mutually occurring disorders of metabolic, environmental, and/or genetic origin. MetS includes obesity, hypertension, insulin resistance, dysglycemia, and dyslipidemia [18,19]. At present, five elementary symptoms are perceived as the markers of MetS. They incorporate an increased waist circumference related to abdominal obesity, high blood pressure, elevated levels of triglycerides (TGs), lowered levels of high-density lipoproteins (HDLs), and high blood glucose levels [20,21]. The presence of at least three of these components defines the existence of metabolic syndrome. The current consensus of several major organizations regarding MetS diagnostic criteria was established in 2009 [22], and it is presented in Table 1. A graphic representation of metabolic syndrome components is shown in Figure 1.

**Figure 2:**
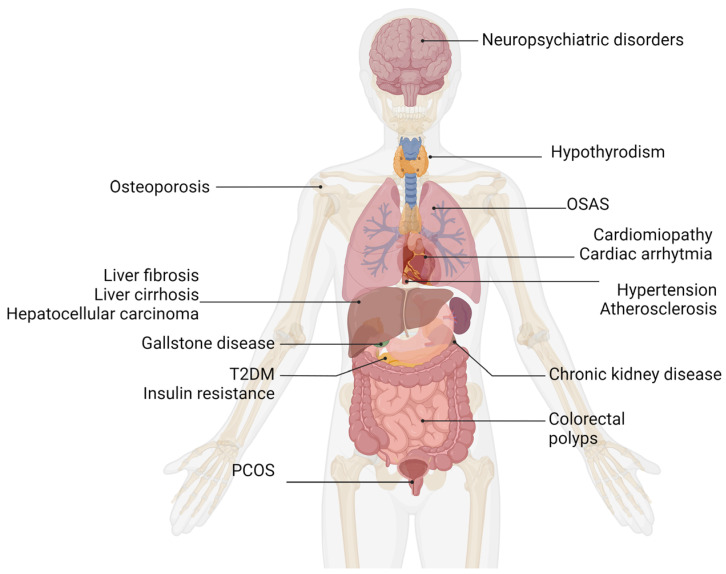
Selected comorbidities connected with MASLD. Adopted and modified from figures in Muthiah et al. [40]. Abbreviations: T2DM—type 2 diabetes mellitus, PCOS—polycystic ovary syndrome, OSAS—obstructive sleep apnea syndrome.

There is growing evidence that MASLD is very closely connected with MetS [23]. Apart from the simple hepatic manifestation of MetS, mainly due to its strong association with abdominal obesity, atherogenic dyslipidemia, dysglycemia, and hypertension, the cause-and-effect relationship between MASLD and MetS is more complex than it was previously thought [24]. Apparently, the main symptoms of MetS, including alterations in blood lipids and glucose levels, can be strongly related to an increased risk of MASLD development, yet this relationship might be bi-directional. In other words, MASLD without other comorbidities can be also a predictor of future manifestation of MetS components [25,26,27]. For example, the results of a meta-analysis by Mantovani et al. show that individuals with MASLD have a 2.2-fold increased risk of incident diabetes [28]. On the other hand, T2DM is deemed as the independent predicting factor of MASLD development and progression [29]. Likewise, a bi-directional relationship has been reported in the case of MASLD and hypertension [30,31], chronic kidney disease [32], or gallstone disease [33]. There are also many reports on MASLD and an increased risk of cardiovascular disease (CVD): CVD events, atherosclerosis, cardiomyopathy, and cardiac arrhythmias [34,35,36,37,38,39]. Apart from CVD or hepatic comorbidities, there is some evidence of other diseases coexisting with MASLD; however, it remains unclear what is the nature of their relationships with liver steatosis [40]. In Section 2.1, certain disorders and conditions related to MASLD pathogenesis are discussed. A schematic representation of selected MASLD comorbidities is shown in Figure 2.

### 2.1. Pathogenesis of MASLD

The exact mechanisms underlying MASDL pathogenesis and progression are still not fully understood, despite significant advancement in recent years. Early theories evolved into the ‘two-hit’ hypothesis of MASLD. They accounted for improper diet, sedentary lifestyle, obesity, and insulin resistance perceived as the primary causes of MASLD, later known as the so-called ‘first hit’. The ‘second-hit’ was the activation of numerous processes in the liver like inflammatory cascades and fibrogenesis [41]. However, recent studies on MASLD cast doubt on this theory, so the ‘multiple-hit’ hypothesis for the progression of MASLD has replaced an outdated presumption [42]. There is growing evidence that MASLD pathogenesis is more complex than previously believed and in the light of novel findings more contributing factors of MASLD development should be taken into account. A brief summary of the currently most probable liver steatosis causes included in the ‘multiple-hit’ hypothesis and related to chlorogenic acid activity is provided in the next subsections.

#### 2.1.1. Insulin Resistance and Alterations in Serum Lipid Levels

The exaggerated intake of food especially rich in fats results in the storage of excess energy in the form of lipids. In physiological conditions, most of the lipids storage takes place in white adipose tissue (WAT), considered as an energy reserve for the organism [43]. In certain conditions, the metabolism of lipids could be impaired and may lead to ectopic fat accumulation, yet MASLD is a model example of this phenomenon [44]. To date, three major reasons for excessive lipids (in the form of triglycerides, TGs) accumulation in the liver have been proposed: improper diet, increased hepatic lipid synthesis, and an exaggerated amount of lipids in WAT [45], which results in the excessive uptake of free fatty acids (FFAs) by liver cells [42]. FFAs originate from dietary products as well as from de novo lipogenesis (DNL) and from the lipolysis of lipids stored in WAT. Alterations in FFA levels in serum lead to excessive TG synthesis in the hepatocytes and their accumulation in liver cells, which results in initial steatosis and could lead to more severe conditions, like liver fibrosis, cirrhosis, and even liver failure [46]. Also, in the course of MASLD alterations in other lipid metabolic pathways are observed. This may lead to an imbalance of the main fractions of serum lipoproteins: very low density (VLDL), low density (LDL), and high-density (HDL) lipoproteins. What is more, the oxidation of FFAs may also be impaired; thereby, the most important pathways of the elimination of hepatic cholesterol and TG can be disrupted in the course of MASLD [46,47]. Moreover, alterations in FFA levels may be caused by improper insulin signaling. Insulin, despite its role in carbohydrate metabolism, has a strong anti-lipolytic effect in the adipose tissue and promotes TG accumulation in WAT. Impaired adipose tissue cells’ response to insulin leads to exaggerated lipolysis [48]. De novo lipogenesis is regulated by a number of transcription factors, with a major role of sterol regulatory element-binding protein—1c (SREBP-1c) and carbohydrate response element binding protein (ChREBP). The activation and regulation of SREBP-1c are mediated by insulin receptor substrate 2 (IRS-2), yet in the course of insulin resistance (IR), this receptor is downregulated, thereby leading to the overexpression of SREBP-1c and the intensification of DNL [42,48]. The activation of ChREBP is glucose-dependent; therefore, the elevated levels of blood glucose in the course of IR intensify DNL. Hence, IR is deemed to be a crucial factor in the development of liver steatosis caused by the excessive influx of FFAs from WAT and the increased synthesis and deposition of TG in hepatocytes [44].

#### 2.1.2. Endoplasmic Reticulum Stress, Lipotoxicity, Inflammatory Processes, and Autophagy

The excessive influx of the FFAs to the hepatocytes leads to the overload of liver cell capacity and results in the impairment of the utilization of FFAs to TG. In a healthy liver, free electrons from oxidization processes in mitochondria are mostly picked up by cytochrome c and finally connected with protons, resulting in a neutral compound—water. However, in MASLD, the excessive quantity of FFAs may disturb the electron transport chain and cause an impairment of the neutralization of reactive oxygen species (ROS), so certain molecules like superoxide (O_2_**^.^**) and hydrogen peroxide (H_2_O_2_) are produced in significant amounts [49]. ROS, in turn, may promote oxidative and endoplasmic reticulum stress, which can result in forming the highly reactive derivatives of FFAs. These compounds may be transported to extracellular space and subsequently induce damage to surrounding tissues [49,50]. Thus, lipotoxicity and related oxidative stress may also activate an inflammatory signaling cascade, resulting in the upregulation and overexpression of numerous pro-inflammatory molecules. They include tumor necrosis factor-α (TNF- α), transforming growth factor-β (TGF- β), interleukins -6 and -1β (IL-6, IL-1β) as well as pro-inflammatory cytokines from the activated pathways of cyclooxygenase (COX) and lipoxygenase (LOX). Hepatocytes, constantly overloaded by the excessive amounts of FFAs, are unable to properly manage ROS, resulting in a vicious circle of chronic inflammation, which is considered as main reason for histopathological and biochemical alterations in the course of MASLD [48,51,52].

Autophagy is an intracellular mechanism whose main function is the maintenance of cell homeostasis by transporting and degrading dysfunctional, damaged, or wasted proteins and organelles [53]. Concurrently, autophagy processes are responsible for the recycling of many small molecules such as proteins, carbohydrates, and lipids, so they can be readily available and reutilized in further metabolic pathways [54]. Lipid droplets present in cytosol are considered as the main cellular reservoir of TG and cholesterol, and they are deemed as typical organelles. Therefore, they should be regulated by standard processes of lysosomal degradation [55]. In physiological conditions, autophagy pathways maintain proper TG hydrolysis to FFAs and help with their efflux from the liver. In patients with MASLD, decreased autophagy impairs the formation and release of TG from the liver [56]. The inhibition of liver autophagy leads to reduced lipid oxidation and an increased accumulation of TG, and in this way contributes to the development of steatosis [57]. Alterations in TG and FFA transport from hepatocytes as well as excessive lipid deposition in hepatic cells lead to the apoptosis of hepatocytes, namely lipoapoptosis [58].

#### 2.1.3. Gut Microbiota

The entire collection of various microorganisms that colonize the human gastrointestinal (GI) tract is called the ‘gut microbiota’. It is composed of bacteria, archaea, viruses, and eukarya, whereas its number may exceed 10^14^ [59]. The gut microbiota plays a crucial role in a wide range of physiological processes, such as regulating immunity, strengthening gut integrity as well as protecting against pathogens and providing nutrients [59]. It has been also found that there is a close relationship and communication between the gut and liver, since the main source of blood for the liver is derived from the portal vein, yet many beneficial substances produced in hepatocytes are absorbed by the gut. This bi-directional communication system was named the ‘gut/liver axis’. Disturbances in one part of this axis may have harmful consequences on the second and vice versa—certain improvements in one organ might cause beneficial effects in the other [60,61].

Intestinal permeability plays a crucial role in determining what substances can pass from the gut into the liver and could lead to the development and progression of MASLD. It relies on the integrity of the intestinal barrier, which includes the mucus layer, intestinal epithelium, mucosal immune system, and the gut vascular barrier (GVB) [62]. Within this barrier, enterocytes and the GVB are primarily responsible for regulating the entry of substances into the portal vein and their subsequent access to the liver [62]. Any disruption of GVB can lead to endotoxins entering from the gut lumen to the liver, resulting in an increased hepatic expression of many pro-inflammatory cytokines and chemokines [63]. Distinct changes in the abundance of certain species in gut microbiota might lead to alterations in the secretion of bacterial metabolites [64]. Numerous bacterial genera of the GI tract have the ability to process complex carbohydrates into secondary metabolites, such as short-chain fatty acids (SCFAs). Among this group, propionate, butyrate, and acetate are perceived as the most important in various cellular processes. The typical ratio of those SCFAs is 1:1:3, respectively [65]. Sufficient and adequate SCFA amount in the gut/liver axis has a beneficial effect in the course of MASLD. Butyrate, for instance, is involved in maintaining appropriate gut microbiota composition, promoting hepatic glucagon-like peptide 1 (GLP-1) receptor expression, and supporting proper intestinal permeability [62]. In turn, the excessive amount of acetate is perceived as obesogenic due to its ability to increase lipogenesis and cholesterol synthesis in the liver and WAT [66]. Thus, dysbiosis in the gut, manifesting as decreasing Firmicutes to Bacterioides ratio and increased acetate synthesis, may promote liver steatosis [67]. Recent studies by Zhou et al. report that gut dysbiosis caused by a high-fructose diet in mice results in alterations in the Firmicutes/Bacterioides ratio and it has been connected with a significantly higher risk of diabetes or obesity. What is more, these changes in gut microbiota may be responsible for the increased expression of many pro-inflammatory molecules (TNF-α, IL-6, and IL-1β) and could result in chronic inflammation [68]. There are also reports on the significance of bile acid metabolism and circulation and their disturbances in the course and development of liver steatosis. Bile acids (BAs) are major components of bile and they are synthesized from cholesterol in the liver via two main pathways [2]. They are stored in the gallbladder and released into the duodenum in response to food intake. Their main function is to emulsify; thus, BAs facilitate the absorption of lipids and water-insoluble vitamins [2]. It has been proven that BAs also serve as signaling molecules, activating FXR and TGR5 receptors. The activation of FXR results in an increased lipid metabolism. Both FXR and TGR5 receptors are also widely expressed in enteroendocrine L-cells, where they stimulate the secretion of GLP-1; thereby, they are indirectly involved in insulin secretion [3]. The study of Ridlon et al. suggests that the Firmicutes phylum has the potential to metabolize primary BAs to secondary metabolites, thus disrupting the FXR and TGR5 signalization pathways and leading to alterations in the metabolism of carbohydrates and lipids [69]. In summary, the role of gut microbiota in liver steatosis is complex and multifaceted. Gut dysbiosis and its related consequences seem to be of the greatest importance in the intestinal pathways of MASLD pathogenesis. A comprehensive review regarding gut/liver axis alteration’s impact on the development of MASLD has been recently published elsewhere [70].

## 3. Chlorogenic Acid Source and Bioavailability

**Figure 3:**
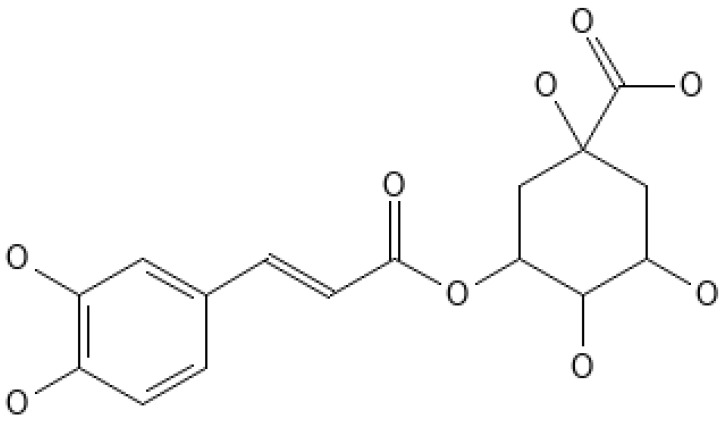
Chemical structure of chlorogenic acid (CGA).

Chlorogenic acid is produced in plants via the chiacid pathway during aerobic respiration. Numerous studies have demonstrated that chlorogenic acid exhibits several key biological activities [71]. These include safeguarding the liver and kidneys, antioxidant properties, regulating sugar and lipid metabolism, anti-inflammatory effects, antimicrobial properties, anticancer effects, and safeguarding the nervous system [71]. The chemical structure of CGA is shown in Figure 3.

CGA is a phenolic compound that is significantly present in green coffee (60 mg/g). In addition to green coffee, fruits, vegetables, and Chinese herbal medicines are also rich sources of chlorogenic acid. For instance, apples contain 0.41–1.16 mg/g of CGA, aubergines contain 1.4–28.0 mg/g, and carrots contain 0.3–18.8 mg/g (DW). Chlorogenic acid is also found in yerba mate, hawthorn, artichokes, stinging nettles, blueberries, and raw potatoes, and in smaller amounts in ivy, plums, cherries, peaches, and apricots [72].

CGA is absorbed in at least two ways. One is immediate absorption in the stomach or upper gastrointestinal tract, and the other is slow absorption in the small intestine. Absorbed CGA is either fully utilized or hydrolyzed to combine with a sulfate, methyl group, or glucuronic acid. It can also undergo hydrogenation, oxidation, or metabolism by the gut microflora [73]. The study found that only 0.8% of the CGA was recovered in urine, and that microbial pathways accounted for 57.4% of the total initial CGA intake, indicating a significant amount of microbial metabolites. This suggests that the bioavailability of CGA is closely linked to the metabolic capacity of the intestinal microbiota [73].

## 4. Biological Activity of Chlorogenic Acid in the Context of MASLD

### 4.1. Antioxidant Effects

Oxidative stress is caused by an imbalance between reactive oxygen species and the antioxidant defense system [71] is endowed with a complex antioxidant mechanism, namely the following:

- Its polyhydroxy structure directly scavenges free radicals and activates the antioxidant signalling pathway;
- CGA regulates the expression levels of related genes and increases antioxidant capacity;
- CGA directly regulates the activity of the endogenous oxidase system and related proteins.

The natural antioxidant activity of CGA depends on its molecular structure, which has five active hydroxyl groups and one carboxyl group. The phenolic hydroxyl structure reacts readily with free radicals and forms free hydrogen radicals with antioxidant activity. This eliminates hydroxyl radicals and superoxide anions, demonstrating strong antioxidant activity [74]. It has been discovered that CGA can inhibit hydroxyl groups from attacking sulfhydryl groups and transform into intramolecular or intermolecular disulfide bonds due to its polyhydroxyl structure. Additionally, CGA can increase the antioxidant capacity of the protein by forming a protein/polyphenol complex through hydrogen bonding with the protein, inhibiting free radical diffusion [68].

### 4.2. Anti-Inflammatory Activity

CGA interacts directly with the NF-κB (nuclear factor kappa-light-chain-enhancer of activated B cells) signaling pathway to regulate the expression of both anti-inflammatory and pro-inflammatory compounds. It comprises structurally related proteins, including p50, p52, p65, c-RelA, and RelB. The activation of NF-κB is facilitated by the phosphorylation of its kinase complex, which includes the IκB restriction subunit. After phosphorylation, IκB is degraded, releasing the p65 subunit of NF-κB [75]. This subunit initiates an inflammatory response by translocating to the nucleus and binding to specific gene promoters of pro-inflammatory proteins. The physiological environment determines the effect of NF-κB on inflammation [71]. The role of CGA is to regulate the expression of proteins and genes involved in the inflammatory response, with the aim of preventing inflammation-induced damage to the body. Furthermore, it has been shown to decrease the levels of tumor necrosis factor-α (TNF-α) as well as the concentrations of interleukins IL-1b and IL-6 [71].

### 4.3. Protective Effect on the Liver

Liver protection by CGA may involve the regulation of enzymes and proteins associated with the oxidation system, thereby limiting oxidative stress-induced liver damage. Additionally, CGA may also protect the liver by controlling cell apoptosis. The accumulation of saturated fatty acids can lead to the apoptosis of liver cells and endoplasmic reticulum stress, which can result in liver fibrosis, hepatitis, cirrhosis, and even liver cancer. In order to prevent this, CGA has been shown to reduce the levels of endoplasmic reticulum stress markers, including glucose-regulated protein 78 (GRP78), C/EBP homologous protein (CHOP), and glucose-regulated protein 94 (GRP94), and prevent the palmitic acid-induced apoptosis of hepatocytes [76]. Additionally, CGA has been found to counteract inflammation and slow the progression of liver disease. In a well-established model of Concanavalin A (ConA)-induced hepatitis, it has been confirmed that CGA can reduce the infiltration of macrophages, neutrophils, and activated CD4 T cells into the liver, as well as reduce ConA exposure [77]. CGA has been shown to regulate various pathways, inhibiting inflammatory cytokine production and oxidative damage, which leads to reduced liver fibrosis and carcinogenesis.

Furthermore, CGA has the potential to alleviate steatosis and hepatitis caused by a high-fat diet. It can also reduce serum aminotransferases and blood lipids while increasing insulin sensitivity. Additionally, CGA has been shown to reverse the activation of the Toll-like receptor 4 (TLR4) signaling pathway and the expression of TNF-α and IL-6 in the liver caused by a high-fat diet. Moreover, CGA has been found to increase *Bifidobacterium* content and decrease fecal *Escherichia coli* content. What is more, CGA was found to increase glucagon-like peptide-1 (GLP-1) levels in the portal vein [64]. The study by Ye et al. shows that CGA can increase the abundance of SCA-producing microorganisms in the gut, mainly *Dubosiella*, *Romboutsia*, *Mucispirillum*, and *Faecalibaculum* in HFD mice [78]. The proper amount of SCAs in the gut is found to be important in the prevention and alleviation of MASLD [62]. What is more, they found that GCA may inhibit metabolic endotoxemia [78]. These findings indicate that CGA provides protection against hepatic steatosis and inflammation caused by a high-fat diet through its anti-inflammatory effects, which are associated with the regulation of the intestinal microflora and increased secretion of glucagon-like peptide-1 [79].

### 4.4. Regulation of Carbohydrate Metabolism

High-carbohydrate and high-fat diets may lead to metabolic endotoxemia and inflammation by inducing the secretion of lipopolysaccharides and increasing chylomicrons in the gastrointestinal tract. This can contribute to the development of obesity, metabolic disorders, and hypertension. Additionally, CGA has been shown to improve insulin sensitivity by acting on the expression of enzymes and genes related to glucose metabolism, which may help mitigate the development of diabetes. Hyperglycemia can lead to oxidative stress, resulting in the production of reactive oxygen species. This can decrease the expression of antioxidant enzymes such as superoxide dismutase 1 (SOD) and superoxide dismutase 2 (SOD2), increase the formation of advanced glycation end products, activate the protein kinase C (PKC)-dependent ERK signaling pathway, and cause liver dysfunction. Studies have shown that CGA can reverse these indicators and have beneficial effects on diabetes [80]. Research has demonstrated that CGA has the potential to reduce fasting blood glucose levels and stimulate insulin secretion in individuals with impaired glucose tolerance, thereby alleviating diabetes [81]. While CGA itself does not impact the phosphorylation of phosphatidylinositol 3-hydroxykinase (PI3K) and insulin receptor substrate (IRS-1), its metabolites can phosphorylate PI3K and IRS-1, leading to increased insulin sensitivity and a beneficial effect on type 2 diabetes [82].

### 4.5. Regulation of Lipid Metabolism

**Figure 4:**
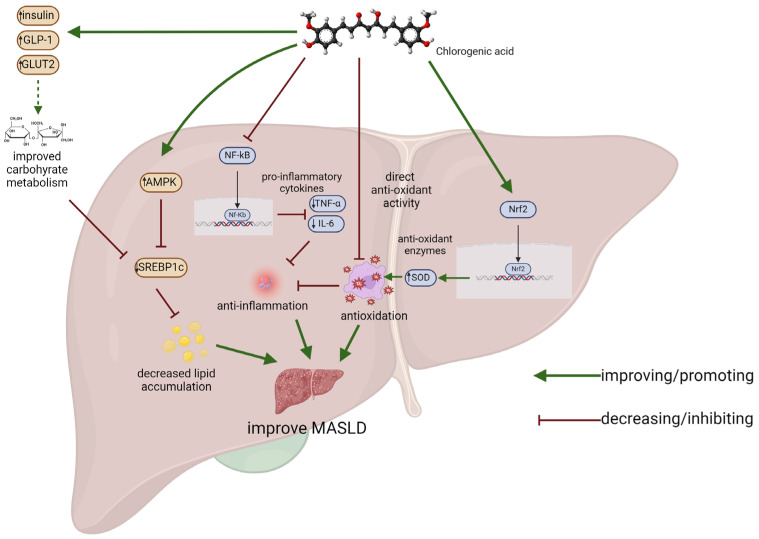
Molecular mechanisms of chlorogenic acid in the improvement of MASLD. For further explanations, please see the text in the Section 4.

The results of research indicate that CGA enhances glucose uptake by increasing the expression of glucose transporter 2 (GLUT2) and phosphofructokinase. This leads to the stimulation of oxidative phosphorylation in brown adipose tissue, resulting in the activation of brown adipose thermogenesis and the release of endocrine fibroblast growth factor-2. Moreover, the combination of chlorogenic acid and caffeine has anti-obesity effects and regulated lipid metabolism induced by a high-fat diet through the AMPKα-LXRα/SREBP-1c (AMP-activated protein kinase α—liver X receptor α/sterol regulatory element binding protein-1c) signaling pathway. CGA regulates the expression of proteins and enzymes that are associated with fatty acid metabolism, thereby reducing the accumulation of fatty acids [83]. The graphical representation of the selected molecular mechanisms of chlorogenic acid mode-of-action in MASLD is shown in Figure 4.

### 4.6. Animal Studies

Chlorogenic acid alone or in combination with various artificial or natural medicines has been widely studied in animal obesity or MASLD models generally showing hepatoprotective properties; however, the molecular mechanisms that underlie alleviating the course of MASLD in rodent models are not entirely clear to date. Significant improvements in liver steatosis, and lipid and glucose metabolism have been observed after CGA administration in animal models. The potential mechanisms are diverse, although the majority of them involve the upregulation of PPARα (peroxisome proliferator-activated receptor alpha) expression, the downregulation of NF-κB activity, the activation of liver autophagy processes, as well as the anti-inflammatory and antioxidant properties of CGA.

Promising effects are reported regarding the gut microbiota and the regulation of the gut/liver axis, especially the role of GLP-1 in MASLD improvement. Research shows that treatment with CGA reduced the levels of Escherichia and increased *Bifidobacterium* in the HFD mice, so CGA may have the potential in maintaining the correct *Bacteroides*/*Firmicutes* ratio in the gut during the course of MASLD. The authors also report improvements in the intestinal mucosal barrier, decreased levels of bacterial lipopolysaccharide (LPS), and increased GLP-1 in the portal vein, suggesting a complex and multifaceted mode of action of CGA in alleviating liver steatosis and regulating the gut/liver axis [67,79]. The alleviation of MASLD symptoms and improvement in the serum lipid profile after the co-administration of indole-3-carbionol (I3C) and CGA has been also reported in the study by Bacil et al. In this study, both diet and chemical-induced MASLD mice models have been employed. The daily co-administration of 5 mg of I3C and 125 mg CGA per kg of body weight in mice resulted in both improvements in the serum lipid profile and increased amount of *Alistipes*, *Allobaculum*, *Bacterioides*, and *Odoribacter* bacteria genera in the gut [84], which may have a beneficial impact on MASLD development [85,86].

The role of CGA in the regulation of autophagy in the course of hepatic steatosis has been investigated by Meng et al. In the mouse model of HFD-induced MASLD, they found that the oral administration of CGA may significantly alleviate MASLD symptoms and reduce hepatic lipid deposition including TG and cholesterol via the activation of liver autophagy processes. Further analysis indicates that CGA can increase the expression of AMPK and ULK1 in murine hepatocytes, thus suggesting its potential to regulate autophagy via the activation of the AXL/ERK/LKB1/AMPK/ULK1 signaling pathway. What is more, the study revealed that CGA specifically inhibits ALKBH1 demethylase, which is involved in various autophagy stages [87]. Yan et al., in a rat HFD-induced MASLD model, found that the intragastric treatment of 50 mg/kg CGA significantly suppressed HFD-induced C-Jun N-terminal kinase 1 (JNK-1) level and resulted in ameliorated insulin resistance and autophagy [14], perceived as major factors in MASLD pathogenesis [42].

The anti-inflammatory effect of CGA plays a significant role in the prevention of MASLD. CGA coupled with geniposide (1.34 mg/kg and 90 mg/per day, respectively) in HFD-induced mice MASLD model significantly decreased the level of stearoyl-CoA desaturase-1 (SCD-1), a key regulatory enzyme in the hepatic DNL pathway [88]; decreased the level of pro-inflammatory cytokines (TNFα, IL-1α, IL-1β IL-6) by the deactivation of Kupffer cells [89]; and had beneficial effects in maintaining the physiological function of intestinal mucosal barrier as well as reducing gut-derived LPS level [89]. Improvement in the mice model of diet-induced MASLD has been observed in the study by Zamani-Garmsiri et al. In the HFD mice treated with 0.25% + 0.02% metformin and CGA in the food, respectively, reduced glucose tolerance as well as the decreased expression of profibrosis-related cytokines, namely α-smooth muscle actin (α-SMA) and TGF-β, the inhibition of gluconeogenesis and the activation of 5′ adenosine monophosphate-activated protein kinase (AMPK) have been found [90]. In the study by Gu et al., CGA was combined with pioglitazone, and it has been reported that this treatment significantly alleviates hepatic inflammation by nuclear factor erythroid 2-related factor 2 (Nrf2) activation; moreover, it upregulates the expression of PPARα. It was proven that CGA may block TLR4 and myeloid differentiation primary response 88 (MyD88), thus potentially ameliorating liver inflammation induced via the LPS-TLR4-MyD88 pathway. Interestingly, no effect on lowering elevated LPS levels has been observed [91]. The study by Alqarni et al. reports the beneficial effect of the combination of telmisartan and CGA due to inhibiting sphingosine kinase/sphingoine-1-phosphate (SphK1/S1P) and TLR4 signaling pathways. Notably, in the last study, the mice MASLD model was obtained by a high-fructose diet versus a high-fat diet employed in previously described papers [92]. In turn, contrary findings have been published in the study by Dungubat et al. The authors utilized different mice MASLD models of choline-deficient, L-amino acid-defined, high-fat diet (CDAHFD). CDAHFD mice were randomized and divided into groups receiving 0.05% caffeine, 0.1% CGA, or placebo and were fed for 7 weeks. In the studied groups, biochemical and histopathological parameters were worsened than in the CDAHFD and control mice [12]. It remains unclear whether the uncommon MASLD model exerted in this study or other factors may have an impact on final results and further studies are required in this field.

A number of reports have indicated that CGA may be effective in alleviating MASLD by reducing the expression of nuclear PPARα. Ding et al. investigated the role of ZFP30 transcriptional factor on PPARα activity and its further role in fatty acids oxidation and subsequent lipid deposition in liver tissue. They employed two independent mice MASLD models: HFD-induced MASLD and ob/ob mice. The intervention was the administration of 90 mg/kg CGA intragastrically for 4 weeks after 12 weeks of HFD or in the 12th week of the experiment in the HFD and ob/ob mice groups, respectively. Liver samples were subsequently collected and analyzed. They found that Zfp30 gene expression was significantly increased in the two MASLD models when compared to the control group. In mice treated with CGA, the deposition of fat droplets in the liver tissue was markedly lower than in HFD or ob/ob mice without intervention. What is more, similar results were obtained in L02 and HepG2 as well as mouse primary hepatocyte cell cultures treated with FFAs. In the CGA-treated mice and HepG2 cells, the expression of ZFP30 was significantly decreased and PPARα expression was successfully restored. These data suggest that ZFP30 may become a potential target for MASLD treatment [15]. Studies have also been published on the beneficial effects on mice and rats of CGA contained in some herbal compositions through the reducing of the expression of PPAR. For instance, *Silphium perfoliatum* L. extract, rich in CGA, regulates the AMPK/FXR/SREPB-1c/PPAR-γ signaling pathway [93], Mailuoning Oral Liquid increased the expression of PPAR gamma coactivator-1α (PGC-1α) and restored the decreased expression of nuclear PPARα [94], yet natural dietary supplement Kèpar^®^ shows not only beneficial effects in MASLD but also in atherosclerosis [95]. Mulberry Leaf Extract (MLE) combined with neochlorogenic acid (nCGA), an isomer of CGA, has been studied by Tsai et al., and found to be an effective treatment in HFD-induced MASLD db/db mice. The study shows significant improvements in serum lipid profile as well as an increased activity of antioxidant enzymes such as SOD, catalase, glutathione peroxidase, and glutathione reductase [96]. Apart from mice MASLD models, in the study by Tie et al., a zebrafish HFD-induced MASLD model has been proposed and employed to investigate CGA and its isomer’s impact on liver steatosis. ROS level has been found to decrease as well as the expression of enzymes involved in lipogenesis such as fatty acid synthase (FAS) and acetyl-CoA carboxylase (ACC). In turn, PPARα expression was found to be increased [97]. The results indicate that CGA and its isomers may have beneficial effects in alleviating MASLD symptoms in diverse animal models, not only in rodents but in zebrafish too.

An epigenetic approach to MASLD has been proposed in the paper by Xu et al. [98], where the authors targeted p300 histone acetyltransferase (p300) as a major epigenetic transcriptional mechanism underlying MASLD pathogenesis. In this study, in HFD-induced obesity rat model, 1 mL/100 g CGA was administered orally to the animals for 4 weeks. Histopathological examination confirms the beneficial impact of CGA in HFD rats. Moreover, CGA was included in the study after the in silico screening of low molecular weight bioactive compounds originating from seaweeds and virtual molecular docking procedure [98]; therefore, this method of screening may be helpful in seeking new anti-MASLD compounds.

## 5. Clinical Trials

Although recent meta-analyses of observational studies have demonstrated a beneficial association between chlorogenic acid-rich coffee consumption and a reduction in the risk of MASLD and liver fibrosis [99], observational studies are unable to provide evidence of a causal relationship. Consequently, clinical trials are necessary to identify a potential cause-and-effect relationship. Table 2 provides an overview of the results of clinical trials conducted to date on the efficacy of chlorogenic acid in the treatment and prevention of MASLD.

The results of the study conducted by Castellino et al. [100] indicated a significant improvement in the metabolic parameters of the liver, with a change in the rate of hepatic steatosis of −17.7% in the group taking the supplement compared to 4.17% in the placebo group. The only reported side effect was transient gastrointestinal symptoms. Mansour et al. [101] conducted a study to determine the efficacy and safety of 6-month supplementation with chlorogenic acid and/or caffeine in type 2 diabetic patients with MASLD. The results of this study indicate that a six-month supplementation regimen comprising two primary coffee components had no discernible impact on the non-invasive markers of hepatic steatosis and fibrosis, liver biochemistry, and inflammatory and metabolic parameters in patients with NAFLD and type 2 diabetes. With the exception of a decrease in the total cholesterol level observed in the caffeine group, and an increase in insulin concentration observed in the chlorogenic acid group with caffeine compared to the placebo group, no statistically significant differences between the groups were observed in any other parameters. Nevertheless, the dosage was found to be completely safe, with no reported side effects. The differences in the results of the two studies by Castellino et al. [100] and by Mansour et al. [101] can be attributed to two factors: differences in the dosage of the active compounds during the two trials and the fact that a correlation between CGA administration and MASLD status can only be observed in certain subpopulations of the patients with MASLD. Further trials with higher doses, longer durations, and different active ingredients in coffee, including chlorogenic acid, are strongly recommended.

Yanagimoto et al. [102] conducted a randomized, double-blind, placebo-controlled crossover study to evaluate the effects of the combined intake of catechins (GCs) from green tea and chlorogenic acids from coffee on postprandial glucose, insulin/incretin response, and insulin sensitivity in eleven healthy men. The results showed that this combination may be effective in preventing diabetes. The findings demonstrated that the continuous, combined consumption of GC and CGA for three weeks suppressed hyperglycemia and insulin levels after consuming a high-fat test meal and improved insulin sensitivity in healthy males. Furthermore, the combined consumption of GC and CGA promoted postprandial GLP-1 secretion and suppressed glucose-dependent insulinotropic polypeptide secretion. This suggests that the increase in GLP-1 may be responsible, at least in part, for the improvements in insulin sensitivity.

The objective of the next clinical trial [103] was to investigate the impact of chlorogenic acid-rich green coffee extract on obesity, glycemic index, and leptin levels in patients with MASLD. The study found that supplementation with chlorogenic acid-rich green coffee extract improved glycemic parameters and adiposity scores. Similar results have been obtained in the study by Verma et al., where CGA-rich green coffee bean extract (CGB70^®^) was investigated in 105 patients with BMI > 25 kg/m^2^ [104]. This suggests that CGA-rich green coffee extracts may be useful in treating the complications of MASLD. These effects appear to be mediated by improvements in insulin resistance, which in turn result in a reduction in fasting blood glucose and obesity indexes in patients with MASLD. Further investigations are required to ascertain the efficacy of chlorogenic acid-rich green coffee extract in the management of MASLD. What is more, more studies are required to ascertain the effects of the extract’s main constituents on the complications of this metabolic disease.

## 6. Conclusions and Perspectives

This review presents a summary of the pharmacological reports on the hepatoprotective effects of CGA. The studies indicate that CGA has promising therapeutic potential in the treatment of various liver diseases, including MASLD and liver fibrosis. The mechanisms of action include antioxidant, anti-inflammatory, and anti-apoptotic effects via the activation of the Nrf2 signaling pathway and the inhibition of the TLR4/NF-κB signaling cascade. Furthermore, the alleviation of liver disease by CGAs also involves other important molecules such as AMPK and important physiological processes such as the intestinal barrier and gut microbiota. It is, therefore, imperative that clinical studies be conducted to confirm the effectiveness of chlorogenic acid in strengthening the intestinal barrier and maintaining a proper gut microbiome.

However, the specific target cell and key molecule targeted by CGAs remain unknown, requiring further in-depth investigation. While some clinical trials have employed nutritional supplements containing CGA and other compounds for the treatment of MASLD, it is strongly recommended that future trials focus on the use of CGAs alone or in combination with other compounds as hepatoprotective agents. Although there is an assumption that CGA could be a safe dietary constituent when taken orally, future research should focus on the pharmacological safety assessment of CGA to confirm the maximum effective and safe doses of CGA in both animals and humans.

Furthermore, the structural modification and optimization of CGA with improved bioavailability should be carried out to identify more effective hepatoprotective compounds. In current research and application, CGA still faces several pressing issues that require immediate attention. These include ensuring stability, addressing potential toxicity concerns, and expanding its range of applications.

## Figures and Tables

**Table 1:** Diagnostic criteria of metabolic syndrome according to the International Diabetes Federation (IDF) and American Heart Association/National Heart, Lung, and Blood Institute (AHA/NHLBI) [22].

	Criteria					Diagnosis of MetS
	WC ^1^	TG	HDL	BP	Fasting Glucose	
Value/ indicator	Europe: men: ≥94 cm women: ≥80 cm United States: men: ≥102 cm women: ≥88 cm Asia: men: ≥90 cm women: ≥80 cm	≥150 mg/dL or treatment for elevated TG *	men: <40 mg/dL women: <50 mg/dL or treatment for reduced HDL *	systolic: ≥130 mmHg diastolic: ≥85 mmHg or treatment for hypertension *	≥100 mg/dL or treatment of elevated glucose *	≥3 criteria

Abbreviations: WC—waist circumference, TG—triglycerides, HDL—high-density lipoproteins, BP—blood pressure, MetS—metabolic syndrome. ^1^ waist circumference as MetS including criterium is population- and country-specific; *—alternative indicator.

**Table 2:** Effect of CGA against MASLD—clinical trials.

Compound	Method	Result	References
CGA and its derivatives + luteolin and its derivatives (Altilix^®^)	6-month,	improved body weight, waist circumference, plasma lipids, hepatic transaminases, flow-mediated dilation, and carotid intima/media thickness	[100]
or placebo	100 patients with metabolic syndrome/MASLD		
	Altilix^®^ group: 50 patients (26 men and 24 women, mean age 63 ± 8 years),		
	placebo group: 50 patients (28 men and 22 women, mean age 63 ± 11 years)		
	6-month,	no significant differences in improved hepatic outcomes compared to placebo (exception: lower total cholesterol in the caffeine group and higher insulin levels in the CGA and caffeine groups)	[101]
200 mg CGA or 200 mg caffeine or 200 mg CGA plus 200 mg caffeine/day	men and women aged 30–53 years with type 2 diabetes and MASLD		
or placebo			
620 mg GC + 373 mg	3 weeks,	improved postprandial glycemic control, GLP-1 response, and	[102]
CGA + 119 mg caffeine/day or placebo	11 healthy men	postprandial insulin sensitivity	
Chlorogenic acid-rich green coffee extract 300 mg BID or placebo	8 weeks,	reduced fasting blood glucose, insulin resistance, weight, waist circumference, and serum leptin	[103]
	48 patients with MASLD, 20–60 years, BMI of 25–35 kg/m^2^.	levels	
	green coffee extract group:		
	24 patients,		
	placebo group: 24 patients		
Green coffee bean extract CGB70^®^ contains 70% CGA 500 mg BID	12 weeks, 105 participants, BMI > 25 kg/m^2^	reduced fasting blood glucose and HbA1c, serum leptin, thyroid-stimulating hormone, waist circumference, and BMI	[104]

## Data Availability

The data presented in this study are available upon request from the corresponding author.

## Abbreviations

AHA	American Heart Association
AMPK	5′ adenosine monophosphate-activated protein kinase
AMPKα	AMP-activated protein kinase alpha
BAs	bile acids
BP	blood pressure
BID	bis in die (twice a day)
CDAHFD	choline-deficient, L-amino acid-defined, high-fat diet
CGA	chlorogenic acid
CHOP	C/EBP homologous protein
ChREBP	carbohydrate response element binding protein
ConA	Concanavalin A
COX	cyclooxygenase
CVD	cardiovascular disease
DNL	de novo lipogenesis
DW	dry weight
FFAs	free fatty acids
FXR	farnesoid X receptor
GC	catechins
GI	gastrointestinal
GLP-1	glucagon-like peptide 1
GLUT2	glucose transporter 2
GRP78	glucose-regulated protein 78
GRP94	glucose-regulated protein 94
GVB	gut vascular barrier
HbA1c	Hemoglobin A1c
HDL	high-density lipoproteins
HFD	high-fat diet
I3C	indole-3-carbinol
IDF	International Diabetes Federation
IL	interleukin
IR	insulin resistance
IRS-1	insulin receptor substrate
JNK-1	C-Jun N-terminal kinase 1
LDL	low-density lipoproteins
LOX	lipoxygenase
LPS	lipopolysaccharide
LXRα	liver X receptor alpha
MASLD	metabolic dysfunction-associated steatotic liver disease
MetS	metabolic syndrome
MyD88	myeloid differentiation primary response 88
NAFLD	nonalcoholic fatty liver disease
NASH	nonalcoholic steatohepatitis
Nrf2	nuclear factor erythroid 2-related factor 2
NF-κB	nuclear factor kappa-light-chain-enhancer of activated B cells
NHLBI	National Heart, Lung, and Blood Institute
OSAS	obstructive sleep apnea syndrome
p300	p300 histone acetyltransferase
PCOS	polycystic ovary syndrome
PGC-1α	PPAR gamma coactivator-1α
PI3K	phosphatidylinositol 3-hydroxykinase
PKC	protein kinase C
PPAR	peroxisome proliferator-activated receptor
ROS	reactive oxygen species
S1P	sphingoine-1-phosphate
SCFAs	short-chain fatty acids
SOD	superoxide dismutase
SphK1	sphingosine kinase
SREBP-1c	sterol regulatory element-binding protein—1c
T2DM	type 2 diabetes mellitus
TG	triglycerides
TGF- β	transforming growth factor beta
TGR5	Takeda G protein-coupled receptor 5
TLR4	Toll-like receptor 4
TNF-α	tumor necrosis factor alpha
VLDL	very low-density lipoproteins
WAT	white adipose tissue
WC	waist circumference
XBP1	X-box binding protein
α-SMA	α-smooth muscle actin
